# Automated Lymph Node Localization and Segmentation in Patients with Head and Neck Cancer: Opportunities and Limitations of Using a Generic AI Model

**DOI:** 10.3390/diagnostics16020355

**Published:** 2026-01-21

**Authors:** Miriam Rinneburger, Heike Carolus, Andra-Iza Iuga, Mathilda Weisthoff, Simon Lennartz, Nils Große Hokamp, Liliana Lourenco Caldeira, Astha Jaiswal, David Maintz, Fabian Christopher Laqua, Bettina Baeßler, Tobias Klinder, Thorsten Persigehl

**Affiliations:** 1 Institute of Diagnostic and Interventional Radiology, Faculty of Medicine and University Hospital Cologne, University of Cologne, 50937 Cologne, Germany; andra.iuga@uk-koeln.de (A.-I.I.); mathilda.weisthoff@uk-koeln.de (M.W.); simon.lennartz@uk-koeln.de (S.L.); nils.grosse-hokamp@uk-koeln.de (N.G.H.); liliana.caldeira@uk-koeln.de (L.L.C.); astha.jaiswal@uk-koeln.de (A.J.); david.maintz@uk-koeln.de (D.M.); thorsten.persigehl@uk-koeln.de (T.P.); 2 Philips Innovative Technologies, 22335 Hamburg, Germany; heike.carolus@philips.com (H.C.); tobias.klinder@philips.com (T.K.); 3 Institute of Diagnostic and Interventional Radiology, RoMed Klinikum Rosenheim, 83022 Rosenheim, Germany; 4 Institute of Diagnostic and Interventional Radiology, University Hospital Würzburg, 97080 Würzburg, Germany; baessler_b@ukw.de

**Keywords:** head and neck cancer, deep learning, artificial intelligence, lymph nodes, computed tomography, staging

## Abstract

**Background/Objectives:** Accurate assessment of lymph nodes is of paramount importance for correct cN staging in head and neck cancer; however, it is very time-consuming for radiologists, and lymph node metastases of head and neck cancers may show distinct characteristics, such as central necrosis or very large size. Here, we evaluate the performance of a previously developed generic cervical lymph node segmentation model in a cohort of patients with head and neck cancer. **Methods:** In our retrospective single-center, multi-vendor study, we included 125 patients with head and neck cancer with at least one untreated lymph node metastasis. On the respective cervical CT scan, an experienced radiologist segmented lymph nodes semi-automatically. All 3D segmentations were confirmed by a second reader. These manual segmentations were compared to segmentations generated by an AI model previously trained on a different dataset of varying cancers. **Results:** In cervical CT scans from 125 patients (61.9 years ± 10.6, 100 men), 3656 lymph nodes were segmented as ground-truth, including 544 clinical metastases. The AI achieved an average recall of 0.70 with 6.5 false positives per CT scan. The average global Dice accounts for 0.73 per scan, with an average Hausdorff distance of 0.88 mm. When analyzing the individual nodes, segmentation accuracy was similar for non-metastatic and metastatic lymph nodes, with a sensitivity of 0.89 and 0.85. Localization performance was lower for metastatic than for non-metastatic lymph nodes, with a recall of 0.65 and 0.74, respectively. Model performance was worse for enlarged nodes (short-axis diameter ≥ 15 mm), with a recall of 0.36 and a sensitivity of 0.67. **Conclusions:** The AI model for generic cervical lymph node segmentation shows good performance for smaller nodes (SAD ≤ 15 mm) with respect to localization and segmentation accuracy. However, for clearly enlarged and necrotic nodes, a retraining of the generic AI algorithm seems to be required for accurate cN staging.

## 1. Introduction

Head and neck cancer accounts for about 5% of all cancer cases, which caused 930,000 new cases and 470,000 deaths worldwide in 2020 [1]. Determination of cN (clinical node staging) status prior to therapy planning is essential, since the decision on neck dissection and adjuvant chemoradiation therapy (CRT) [2] has a significant impact on survival: for solitary lymph node (LN) metastasis, 5-year survival is about 50%, whereas it drops down to 33% through involvement of contralateral LN metastasis and even lower in distant metastasis [3,4,5].

In clinical routine, contrast-enhanced CT (CECT) or magnetic resonance imaging (MRI) of the cervical region is performed in most cases at the time of diagnosis, where CT shows a higher specificity to detect LNs than MRI [6]. LNs are typically measured in the short axis diameter (SAD, in mm) on the axial plane for non-invasive characterization of suspicious metastasis. According to the Response Evaluation Criteria in Solid Tumors 1.1 (RECIST 1.1), LNs with a SAD of ≥15 mm are considered as metastatic target lesions, LNs of 10–14 mm as metastatic non-target lesions, and LNs with a SAD ≤ 9 mm as non-metastatic [7]. In the literature, a broad number of different threshold values is described for physiological LNs dependent on the LN level and the patient age [8,9]. However, as recently reported in the lymph node reporting and data system (Node-RADS v1.0) [10], size characteristics alone do not seem to be a sufficient tool to predict metastatic disease correctly; instead, there are several additional morphological aspects to be considered. Majumdar et al. [11] found that 9% of the patients were upstaged after serial sectioning and immunohistochemistry because of micrometastases, and for several LN levels, a threshold of 5 mm was found to be more predictive [10]. In addition to size, other aspects such as texture, border, and shape need to be considered: Nodal metastases in head/neck cancer tend to necrotize more often, with central necrosis having a specificity of 95% to 100% [3,12,13]. In some subtypes of head/neck cancer, such as human papillomavirus (HPV)-associated Head and Neck Squa-mous-Cell Carcinoma (HNSCC) [14], metastatic LNs can also appear as cystic lesions. Moreover, LNs in HNSCC or papillary carcinoma may be (partially) calcified and should not be mistaken for post-inflammatory calcifications common after healed tuberculosis for example [15].

There is also evidence that the number of affected LNs should be monitored: Patients with six or more affected LNs can benefit from postoperative CRT, whilst for patients with a lower nodal tumor burden, postoperative CRT does not improve overall survival when compared to postoperative radiation therapy alone [16]. In HNSCC, the LN ratio, so the amount of metastatic LNs amongst the total amount of LNs, seems to be of prognostic importance as well [17,18]. However, a study conducted in the Netherlands found that the absolute amount of positive LNs tends to be of higher relevance than the LN ratio [19].

Radiomics is a promising method for extracting additional information from medical images to improve the characterization of different tissues, including tumor tissue and LN metastases [20,21,22]. Recent studies have shown the potential of differentiating inflammatory lymphadenopathies from metastatic LNs [23]. So, radiomics might improve the detection of otherwise occult (micro-) metastatic nodules. However, to perform radiomics analysis, there needs to be a robust segmentation of the LNs, of which there can be up to 300 in the head/neck region [24]. To make segmentation more time-efficient, we have developed an AI algorithm for the localization and three-dimensional segmentation of LNs in the head and neck region independently of the underlying pathology.

In this study, we applied our pre-existing AI model to the localization and segmentation of LNs in CECTs of the head and neck region26 to CECTs from patient with head/neck cancer bearing at least one metastatic LN to check its performance on LN metastases of head and neck cancer to see if this algorithm can also segment these specific metastases with the peculiarities described above.

## 2. Materials and Methods

In this IRB-approved study, patient consent was waived due to the retrospective design of the study based on pre-existing images by the Ethics Committee of the Faculty of Medicine, University of Cologne, reference number 19-1390/7 August 2019. We received a second one, which also states that patient consent was waived due to the retrospective design of the study based on preexisting images, on 16 August 2019 (reference number 19-1379). 

### 2.1. Acquisition of the Dataset

CECT scans of the head and neck region from patients, who presented at the department of otorhinolaryngology with either confirmed tumor of the cervical region or suspicion for malignancy between January 2000 and 2021, were searched in our picture archiving and communication system (PACS, IMPAX EE, Agfa HealthCare). We included venous CECT scans conducted from the skull base to the lung apex with a slice thickness between 0.75 and 2.5 mm in patients aged ≥18 years with at least one untreated LN metastasis confirmed through PET/CT-positivity, positive histology, progression/regression under chemotherapy in a follow-up scan, or tumorous central necrosis. We excluded scans with primary tumors of the skin, Hodgkin’s or Non-Hodgkin’s Lymphoma, unclear lesions suspecting soft tissue metastasis, or lymphadenopathy without clinically confirmed metastasis through the criteria mentioned above, CECT scans from patients with a secondary metachronous tumor, or follow-up scans.

In total, we found 126 patients. One had to be excluded because histology confirmed a Warthin’s tumor.

All scans were conducted supine in cranio-caudal direction after a bolus injection of 80 mL of iodinated contrast agent (Accupaque, 350 mg/mL; General Electric Healthcare, Chicago, IL, USA) with an injection rate of 3.5 mL/s and a delay of 40 s after reaching a threshold value of 150 Hounsfield Units (HU, scale used for the quantification of radiodensities in CT scans) in the descending aorta. The scans were conducted on a dual detector layer CT (IQon, Philips Healthcare, Best, The Netherlands) with a tube voltage 120 kV, mostly tube current modulation with a reference of 129 mAs (DoseRight, Philips Healthcare), collimation of 64 × 0.625 mm, and a pitch of 1.23, or on the single-energy CTs (Philips Brilliance 64), or iCT 256 (Philips Healthcare) with a tube voltage of 120 kV, or on Siemens SOMATOM Definition Flash (Siemens Healthineers, Forchheim, Germany) with a tube voltage of 100 kV. One CT scan was conducted on Siemens SOMATOM Force with a tube voltage of 90 kV.

### 2.2. Manual Lymph Node Segmentation

Volumetric ground-truth segmentations for all cervical LNs ≥ 5 mm were generated by one radiologist with more than four years of experience in CT imaging using the 3D multi-modal tumor tracking tool MMTT (IntelliSpace Portal, Version 12, Philips Healthcare) integrated in a dedicated research platform (IntelliSpace Discovery, ISD, Version 3.0, Philips Healthcare). To decrease the annotation effort, a preliminary AI model for LN segmentation was applied to the images, which was the starting point for the annotations. The radiologist reviewed these segmentations by removing false-positives, segmenting nodes missed by the algorithm (false-negatives), and correcting the shape of the segmentations. Since in the head and neck region, neighboring structures such as vessels can complicate LN identification and boundary delineation, these corrections were not only performed in the axial plane but also checked in the coronal and sagittal planes. Segmentations were then double-checked and corrected by a second independent radiologist with more than 16 years of experience in CT imaging when in doubt. Please note that the model used to support the annotation effort is a predecessor of the model used for the evaluation in this study, but it is less sophisticated, especially with respect to augmentation parameters. This might introduce a slight bias towards the AI model. Nevertheless, the results were thoroughly reviewed and adapted by the annotating radiologist to minimize this bias.

### 2.3. Labeling of LN Metastases

For each patient, a histological report, either after fine needle aspiration or after LN extirpation, was searched for in the electronic patient record system (ORBIS, DH Healthcare GmbH, Bonn, Germany) of the institution. Moreover, PET-CT scans of the same patient conducted in a timeframe of 3 months before or after the CECT scan to correlate PET-positive LNs and follow-up CECT scans to assess response or non-response to applied chemotherapeutic agents were searched for in the PACS to identify metastases.

Subsequently, all labeled LNs were reassessed by the two radiologists in consensus and centrally necrotic LNs, PET-positive LNs, histologically metastatic LNs, and LNs with response to chemotherapy were marked as metastatic.

### 2.4. AI-Based Lymph Node Segmentation

A foveal net (f-net) [25] architecture was chosen for the 3D convolutional neuronal network [26], which is available as a research prototype in IntelliSpace Discovery (Version 3.0, Philips Healthcare). This network was specifically trained for LN identification and localization in the head/neck region on 187 CECT scans collected from patients with various pathologies, among others with breast cancer, lung cancer, malignant melanoma, and lymphoma. For more details on the training setup, we refer the reader to [26]. In the initial training dataset, the average SAD was 6.9 mm with a maximum of 44.9 mm. On a similar test dataset with 30 CECT scans, this network achieved an average recall of 0.67 at 8 false positives (FPs)/scan with a global Dice of 0.81.

### 2.5. Evaluation Criteria

The LN software (research prototype, version 1.0) returned a binary mask representing the LN tissue. To yield individual LN instances, a connected component analysis was executed as a post-processing step. Please note that touching LNs, which occurs for some patients due to bulky disease, are still represented as one instance. Predictions with a SAD < 4.5 mm were removed from the prediction mask as they were considered too small.

To assess the localization performance of the network, recall, precision, F1-score, and number of false-positives per volume were computed. An LN was considered localized if the sensitivity, i.e., the ratio of correctly segmented tissue and volume of the LN, was greater or equal to 0.4. Another group [27] proposes using instance over union as the metric to identify localized nodes, but as some of the LNs cannot be matched unambiguously when bulky disease is present, this measure is not feasible here.

The segmentation performance was analyzed, on the one hand, on a global level using the Dice coefficient and the average Hausdorff distance. As we wanted to clearly assess the segmentation quality without biasing it by false positives (FPs) and false negatives (FNs), these measures were computed on masks where FPs and FNs were filtered out.

On the other hand, to analyze the segmentation quality per LN, the sensitivity was computed for each LN. As LNs cannot be matched unambiguously in some cases, this measure is less biased by the LN clusters than the Dice coefficient while still yielding a good impression of how well a node is segmented.

For a more detailed evaluation, LNs were divided into SAD-dependent groups: 5–9 mm, ≥10 mm and ≥15 mm, according to RECIST 1.1 with LNs < 10 mm as physiological or unclear, ≥10 mm as suspected metastasis, and ≥15 mm as metastatic target lesions [7]. In addition, the results were compared between the metastatic and non-metastatic LNs.

### 2.6. Statistical Analysis

Statistical analysis was performed using Python (3.12.7) with the SciPy Stats package (version 1.13.1). The data were split into two groups, namely metastatic and non-metastatic nodes. Due to the lack of normality of the data, which was tested using the Shapiro–Wilk test (*p* << 0.01), a Kruskal–Wallis analysis was performed to compare the recall and sensitivity for the two groups. Kruskal–Wallis analysis is a non-parametric test, which excels as an option for comparing medians when normality fails. The significance level was set to α = 0.01. Pearson correlation analysis was performed to assess the relationship between the short-axis diameter (SAD) of LNs and the segmentation accuracy measured by sensitivity.

## 3. Results

### 3.1. Study Population

In total, CECT scans from 125 patients were included. Further, 100 of these patients (i.e., 80%) were male. The average patient age was 61.9 ± 10.7 (SD) years (females 60.9 ± 11.9 years, males 62.2 ± 10.4 years).

### 3.2. Manual Lymph Node Segmentation and Labeling of Metastases

**Figure 1:**
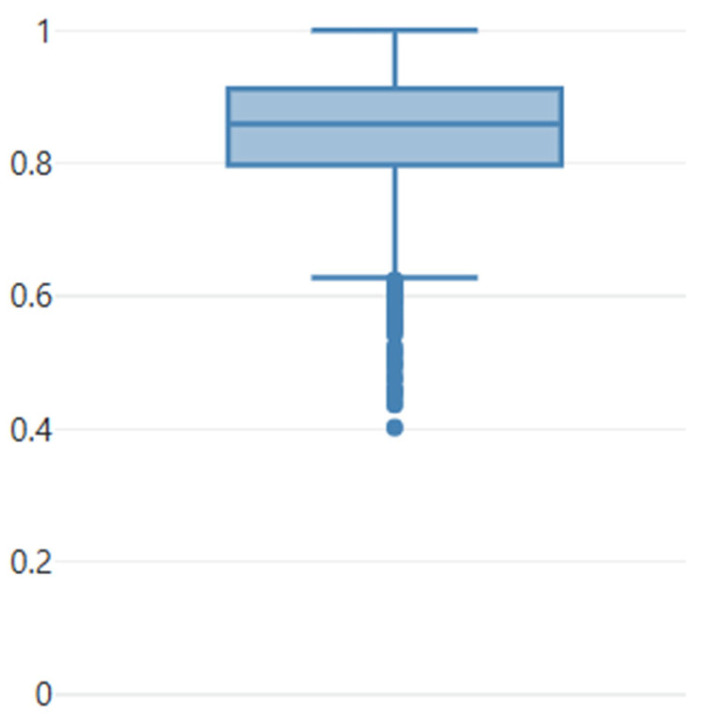
Boxplot showing the sensitivity of the individual nodes computed for the initial predictions compared with the corrected predictions used as ground-truth. As can be seen from the boxplot, almost all predictions underwent a correction. Median sensitivity is 0.86. The dots represent the outliers.

The preliminary model, used to support the manual segmentation, identified 3798 LNs in the 125 CT scans. Of these, 868 LNs were rejected by the annotating radiologist as being too small (less than 4.5 mm) or as real FPs, and 726 missed LNs were additionally segmented. This yields 3656 LNs that were segmented as ground-truth, i.e., an average of 29.2 LNs per patient. The shape of the segmentation was corrected for almost all LNs (namely 99.0%), even if only minor adjustments were needed to achieve the highest segmentation quality, with the median sensitivity between the initial predictions and the ground-truth annotations accounting for 0.86 (for more detail on a per-LN level, see Figure 1).

**Figure 2:**
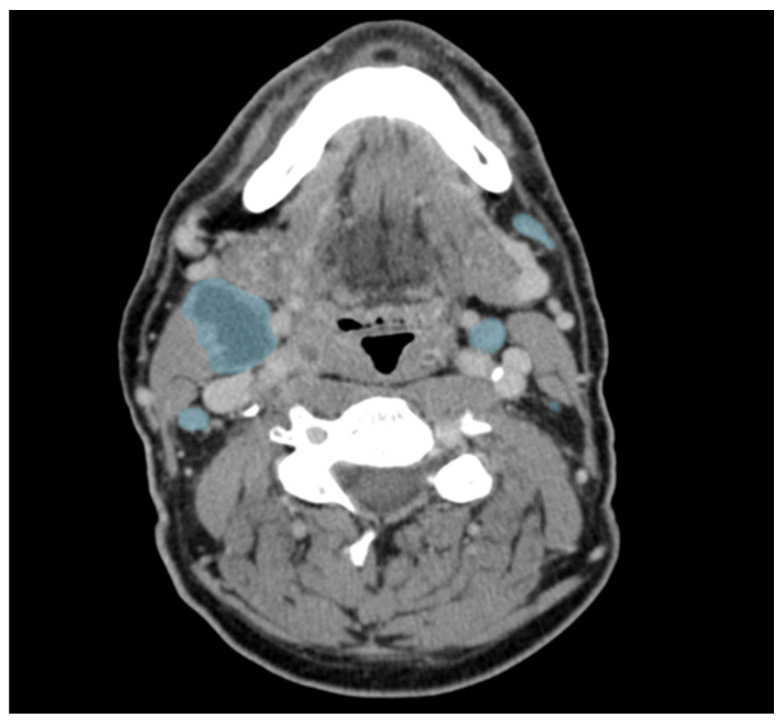
Example of manually corrected lymph node segmentations in an axial contrast-enhanced CT of a patient with head and neck cancer. Lymph nodes are marked in blue. Please note the central necrosis (more hypodense areas) of the large lymph node on the patient’s right.

Figure 2 shows an example of the manual segmentation in one CT slice. In comparison to the corrected ground-truth, the preliminary model achieves a recall of 0.66 with a global Dice of 0.74.

**Figure 3:**
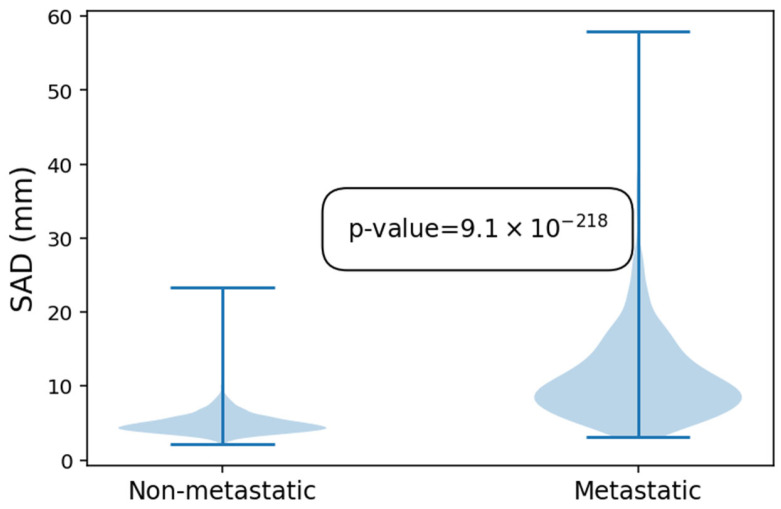
Violin plot showing the difference in size of the non-metastatic and metastatic lymph nodes.

In a second step, 544 of these LNs were marked as metastatic. Non-metastatic LNs had an average SAD of 5.0 mm with a maximum of 23.3 mm, while metastatic LNs had an average SAD of 11.3 mm and a maximum of 57.9 mm. This difference was statistically significant (*p* < 0.01 (9.1 × 10^−218^)). The size distribution amongst metastatic and non-metastatic LNs is further illustrated in Figure 3.

### 3.3. Runtime

Network inference included pre- and post-processing runs for approximately 6 ± 1.3 s on an Nvidia GeForce GTX 1080 GPU for the head-neck CT series from our dataset.

### 3.4. Global Performance

The model achieves an average recall of 0.70 at 6.5 FPs/scan. In general, the FPs are rather small, with an average size of 5.3 mm. When only looking at FPs with a significant size, i.e., ≥10 mm, we arrive at 0.3 FPs/scan. The precision and F1-score are 0.73 and 0.71, respectively.

With respect to the segmentation accuracy, the model achieves a global Dice score of 0.73, with an average Hausdorff distance of 0.9 mm.

**Figure 4:**
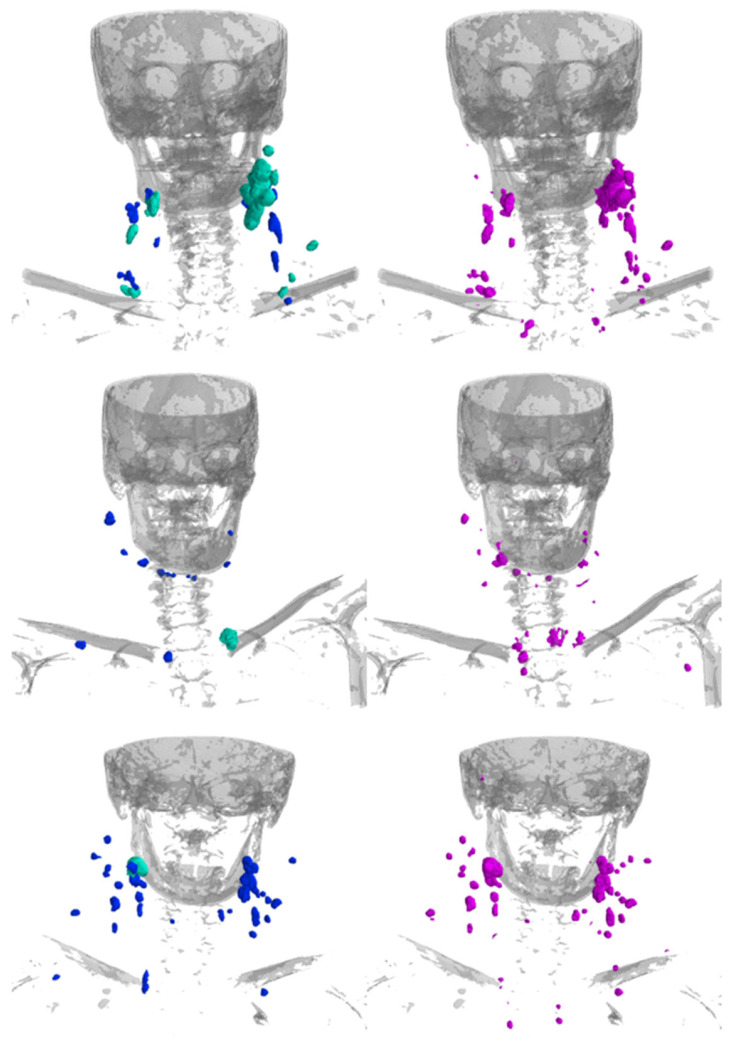
Example renderings of the LNs showing the manual segmentations (**left**; blue = non-metastatic, cyan-green = labeled as clinically metastatic) and the segmentations predicted by the model (**right**; magenta). Bones are rendered in gray for anatomical orientation.

Example cases for visual comparison of the predicted and manual segmentations can be seen in Figure 4. The visual inspection shows good performance independent of the location and size of the LNs.

### 3.5. Performance Relative to Nodal Status

After evaluation of the network performance on a CT series level, the following section will have a more detailed look at the potential differences in performance regarding LNs marked as clinically metastatic and LNs not labeled as a nodal metastasis.

A sub-analysis (see Table 1) showed that the model segmented 354 of the 544 metastatic LNs, yielding a recall of 0.65 for metastatic LNs. For non-metastatic LNs, the recall is 0.74. The Kruskal–Wallis test revealed statistically significant overall differences among the groups (*p*-value = 5 × 10^−6^). Post hoc pairwise comparisons showed that only the <5 mm group differed significantly from the others (*p*-value = 7 × 10^−6^), whereas differences among the 5–9 mm, 10–14 mm, and > 15 mm groups were not statistically significant (*p*-value = 0.03, 0.68, and 0.19, respectively).

On a per-LN level, the average segmentation sensitivity was 0.85 for metastatic and 0.89 for non-metastatic LNs. The Kruskal–Wallis test indicated no statistically significant differences among the two groups (*p* = 0.014).

### 3.6. Performance Relative to SAD

Table 1 details recall and sensitivity with respect to SAD. Here, it is noteworthy that there is a statistically significant difference in the recall of LNs measuring less than 15 mm in SAD compared to those measuring more than 15 mm (*p*-value = 9 × 10^−20^). Specifically, recall is lower for LNs larger than 15 mm, and this pattern holds true for both non-metastatic and metastatic LNs (*p*-value = 0.003 and 3 × 10^−12^, respectively). However, please note that the recall of 0% for non-metastatic LNs > 15 mm may largely be due to the small sample size of 3 LNs.

### 3.7. Segmentation Analysis

When further analyzing sensitivity in relation to SAD, there is a moderate negative correlation between sensitivity and SAD for all LNs (Pearson correlation R = −0.18, *p* << 0.01), as shown in Figure 4. When considering only LNs with a SAD of greater than 10 mm, this correlation becomes stronger (R = −0.37, *p* << 0.01). For SAD values above 10 mm, the sample is dominated by metastatic LNs, with only 14 of 275 LNs being normal. This evidence suggests that larger LNs are segmented less accurately than smaller ones.

**Figure 5:**
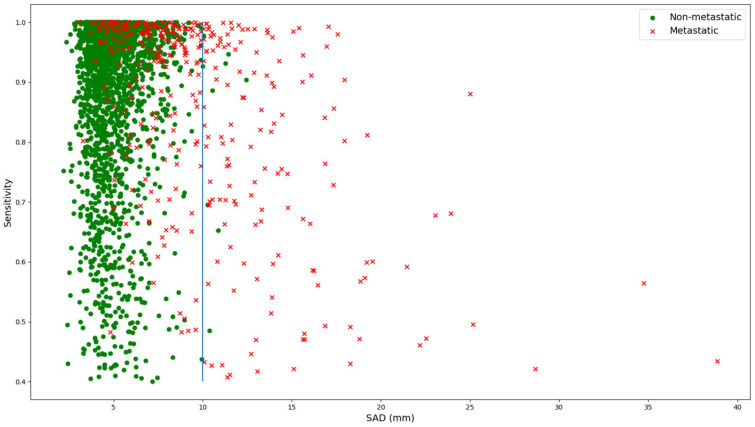
Relation between SAD and sensitivity for both normal (green dots) and metastatic (red crosses) lymph nodes. The blue line marks the threshold of 10 mm as an SAD for lymph nodes, which is the threshold for metastatic lymph nodes according to RECIST 1.1.

To obtain a better visual impression of the segmentation accuracy of both smaller and larger LNs, the radiologist looked at an overlay of the CT scan with both the manual segmentations and the segmentations performed by the AI model (see Figure 5).

**Figure 6:**
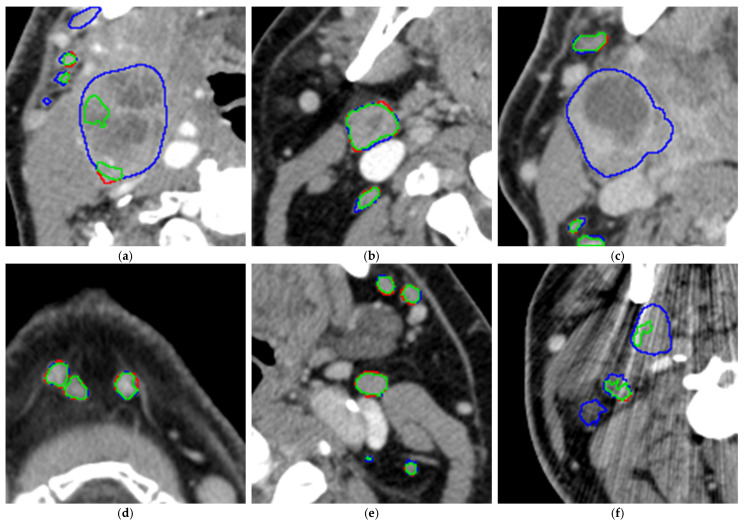
(**a**–**f**). Analysis of the segmentation overlays on 6 example CT slices (green: correct segmentations, blue: false-negative segmentations, thus manually segmented areas left out by the model, red: false-positive segmentations, thus areas segmented by the model but left out in the manual segmentations).

The model tends to leave out the necrotic parts of the metastatic LNs (please see Figure 6a,c), especially for large LNs, while for smaller LNs, it is often able to segment the necrotic center (see Figure 6b). For smaller LNs, the segmentations are accurate (see Figure 6d,e). In addition, metal artifacts from dental implants may affect segmentation accuracy (see Figure 6f) and thus recall.

## 4. Discussion

Overall, our generic cervical lymph node localization algorithm showed an average recall of 0.70, and the average global Dice amounts to 0.73 per scan with an average Hausdorff distance of 0.88 mm. Recall correlated with the SAD of the LNs, but for LNs > 5 mm, there was no difference in recall for metastatic and non-metastatic LNs. On a per LN level, segmentation sensitivity was equal for non-metastatic and metastatic LNs, with 0.89 and 0.85, respectively. But the correlation to the SAD demonstrated that the model had problems segmenting larger LNs. Visual analysis of the AI segmentation of these larger LNs revealed that the model especially tends to leave out the necrotic areas of LN metastases.

Our results for the recall were similar to the ones in the initial test dataset, where recall was 0.67 [26]. However, sensitivity was 0.82 and therefore slightly higher than in this cohort. This might be due to the more homogeneous and challenging nature of this cohort consisting only of patients with head and neck cancer, whereas the initial cohort the model was trained on was very heterogeneous and contained scans with various indications, including different cancer entities and non-cancer pathologies.

Looking into the details, the segmentation sensitivity seems to correlate with SAD. The reason for this might again be that the current model has not learned to identify large LNs, so it might be hesitant to segment these and does so only partially. Moreover, the model tends to leave out the necrotic areas. The initial training cohort was very heterogeneous with respect to pathologies. However, LN necroses are specific for HNSCC but only occur sporadically in lymphomas [3,28] and were therefore not well represented in the initial training dataset. With a portion of 9.6% HNSCC patients and 36.9% lymphoma patients (+6.4% leukemia) in the initial training dataset (for more details see [26]), the model is not specifically trained on LN necroses. So, future work should concentrate on extending the training dataset by increasing the number of scans containing necrotic LNs and by addressing to what extent these challenges might be cancer-type specific.

In a recent study, Ariji et al. [27] trained the neural network ‘DetectNet’, a 2D approach for object detection, on the localization of cervical LNs of patients with oral squamous cell carcinoma. The network reached a recall of 0.73 for metastatic and 0.525 for non-metastatic LNs. This is partially lower than our recall, but it is of note that they calculated recall with a 0.6 overlap compared to our definition of recall as a 0.4 overlap, so that the results are not directly comparable, and it is of note that they had a lower number of included LNs, with only 159 metastatic and 517 non-metastatic LNs of 56 patients in 365 CT images. Additionally, it should be noted that they only used selected CT images and not CT scans. Liao et al. [29] trained an nnUnet on a broader cohort of 626 patients with head and neck cancer. Here, they reached a substantially lower recall of 0.457 to 0.546, depending on the test cohort they used.

In our cohort, recall for non-metastatic LNs initially seemed to be lower than for metastatic LNs. However, when analyzing the LNs > 5 mm, there was no significant difference in the recall of the two groups. A slight non-significant difference remained, which may have resulted from the moderately larger size of the metastatic LNs or, less likely, due to their anatomical location. Metastatic LNs most commonly occur at level II, III, or IV [30]. Missed non-metastatic LNs are often found near the chin or clavicles, which were underrepresented in the training data compared to other levels. At present, we do not have any distribution patterns for the LNs among the cervical LN levels available for this cohort, which future research should concentrate on.

Regarding Global Dice, our model reaches a global dice of 0.73/scan, which is on par with previous publications [26].

When comparing our segmentation accuracy to the recent literature, mostly similar results have been reported: The group of Liao et al. [29] reached a Dice of 0.72 to 0.74 in their dataset, which seems comparable to ours, but again, it is of note that their localization rate is a lot lower than ours. Another group achieved a Dice of 0.81 in the segmentation of cervical LNs [31]. However, it is of note that they did not concentrate on patients with head and neck cancer and they excluded any patient with any known or suspected primary malignancy and any suspicion of nodal spread. They only included physiological, non-enlarged LNs, which tend to be separated from each other, without any touching border, and with a more homogeneous density and central necrosis. In contrast, we investigated mostly enlarged LNs touching each other, which are therefore less distinguishable, as well as LNs with nodal necrosis, which makes the dataset a lot more heterogeneous and influences the Dice. In the MRI-based automated segmentation of cervical LNs, depending on the AI model used, another group reported comparable segmentation accuracies of 0.7412, 0.6223, and 0.7404 [32]. However, it is also of note that they explicitly excluded any touching of LNs. They segmented single LN metastases only and did not perform a complete segmentation of the whole CT scan, as we did.

When comparing our results to deep learning-based segmentation studies on LN metastases in CT scans of patients with another primary tumor and consequently another anatomical region, slightly higher results are reported: Tan et al. [33] concentrated on LNs in patients with colorectal cancer and reached a Dice of 0.832. However, they conducted their study on 54 selected LN in scans of 48 patients only, with an average size (long axis) of 28.00 ± 13.2 mm. It is also of note that for these results, they performed an initial segmentation step by dynamic programming and then added two additional segmentation steps to correct the initial result through morphological smoothing and active contours. Adding additional techniques could therefore also be considered to improve our AI model.

Our population consists of markedly more men than women. This is consistent with an epidemiological study [34] which found that the head and neck cancer incidence rate was about three-fold higher for males, independent of the patients’ smoking and drinking behavior.

We did not exclude CECT scans containing artifacts from dental amalgam because they are present in a huge subgroup of head and neck squamous cell carcinoma.

Another cohort of HNSCC patients, those in a retrospective study, showed that 73.6% of scans had these artifacts [35]. However, these artifacts may mask suspect LNs and impede segmentation.

A limitation of this study is that only one radiologist performed the initial segmentations based on pre-segmented images. Nonetheless, this first radiologist refined most of the pre-segmentations and, whenever there were doubts regarding a segmentation, the results were reviewed by a second radiologist with more than 16 years of experience to guarantee a high data quality. However, in a subsequent study, inter-reader and intra-reader agreement on LN segmentation could be investigated.

Another limitation is that an initial set of ground-truth segmentations was predicted by a preliminary AI model. Nevertheless, the predictions were thoroughly reviewed by adding missed LNs, removing false-positives, and adapting the shapes of the predicted LNs. Throughout this thorough review, we tried to minimize the bias introduced by using the AI model.

Moreover, this study was conducted on CT scans from only one institution, but from two different vendors and different CT scanner types. The inclusion of further (international) centers and extension to scanners from other vendors should further improve generalizability and applicability in clinical practice.

However, it is of note that the pre-existing AI model was trained on one scanner only (IQon, Philips Healthcare, Best, The Netherlands), whereas here, multiple CT scanners were included, and the AI model also had good localization performance on the other scanners.

Since the pre-existing model was only trained on LNs ≥ 5 mm, micrometastases might escape this algorithm. This is a frequent problem in imaging, especially for head and neck cancer. About 50% of LNs harboring malignant cells measure less than 5 mm, and 45% of histologically proven LN metastases with extranodal involvement are not being identified on CT scans [36]. This underlines the need for radiomics analysis than looking at size only.

So, further studies should focus on a subanalysis of different LN levels, with some areas more likely harboring smaller LN metastases and imaging texture features, as reflected by Node-RADS version 1.0 [10]. Moreover, they should focus on the change in size in follow-up scans (delta imaging biomarker). Combined with the location of the LNs, this might help predict survival after CRT [37].

Regarding clinical routine, looking for and assessing smaller LNs is especially challenging and can make cN staging a very time-consuming task. Since our model has a recall of 0.79 for LNs with a SAD of 5–10 mm and runtime is 6 ± 1.3 s for a head and neck CT scan, even at this point in development and despite its weaknesses concerning larger LNs, an implementation of an automated LN segmentation through our model provided to the reporting radiologist as additional information along with the CT images may both speed up and improve the diagnostic confidence of cN staging in clinical practice.

Moreover, the manual segmentation of LNs in a whole head and neck CT dataset can take several hours per patient, making it unfeasible in clinical practice. However, to perform a radiomics analysis, which could provide additional information on the CT data, robust segmentation is absolutely required.

Thus, despite its limitations at this point in time, only an implementation of such an automated model, possibly also as a starting point for further manual corrections, can open the way to integrating radiomics as an additional diagnostic tool in a routine staging.

## 5. Conclusions

In conclusion, our generic cervical LN localization and segmentation AI algorithm is capable of specifically localizing and robustly segmenting most LNs of patients with head and neck cancer. Our results are mostly comparable to and partially better than those in the recent literature on head and neck cancers.

However, segmentation performance is still challenging in the cervical area compared to other anatomical regions with more distinct borders than in the head and neck region, where contact to neighboring structures through marginal surrounding fatty tissue and touching LNs complicate segmentation. Our AI algorithm revealed limitations in segmenting enlarged LNs and especially necrotic areas of metastatic LNs, which is a common feature in LN metastases of head and neck cancer, but uncommon in most other solid cancers and lymphoma. The performance was not perfect in the enlarged and necrotic LN metastases, but these are easy to spot and diagnose in clinical routine and are not the ones causing headaches, different from the small micrometastatic ones. Here, our AI model could help to segment a high number of smaller LNs for more in-depth radiomics analysis, which would not be possible with manual segmentation.

In summary, our study demonstrates that a generic AI model for automatic lymph node localization and segmentation may present limited performance in a specific cancer type. While the generic AI model already has its benefits, a model tuned to a specific cancer type, via a more heterogenous training dataset, might show better performance.

## Figures and Tables

**Table 1:** Total LN number, recall and sensitivity with respect to SAD for non-metastatic (non-mets), metastatic (mets) and all lymph nodes.

		<5 mm	5–10 mm	10–15 mm	>15 mm	All
Number of LNs	all	1906	1474	159	117	3656
	non-mets	1869	1228	12	3	3112
	mets	37	246	147	114	544
Recall	all	0.72	0.79	0.67	0.36	0.73
	non-mets	0.71	0.80	0.67	0.0	0.74
	mets	0.89	0.74	0.67	0.37	0.65
Sensitivity	all	0.88	0.89	0.80	0.67	0.88
	non-mets	0.88	0.89	0.81	-	0.89
	mets	0.94	0.90	0.80	0.67	0.85

## Data Availability

The data presented in this study are available on request from the corresponding author due to privacy restrictions, legal und ethical reasons.

## Abbreviations

The following abbreviations are used in this manuscript:

AI	Artificial Intelligence
CECT	Contrast-enhanced CT scan
cN	Clinical node staging
CRT	chemoradiation therapy
CT	Computed Tomography
Dice	Dice coefficient
DL	Deep Learning
f-Net	Fully Convolutional Network
FP	False Positive
GPU	Graphics Processing Unit
HNSCC	Head and Neck Squamous-Cell Carcinoma
HPV	human papillomavirus
HU	Hounsfield Units
LN	Lymph Node
MRI	Magnetic Resonance Imaging
N-status	nodal status
Node-RADS	Lymph Node Reporting and Data System
PACS	picture archiving and communication system
PET	Positron Emission Tomography
RECIST	Response Evaluation Criteria in Lymphoma
SAD	Short-Axis Diameter
TP	True Positive
US	ultrasound

## References

[1] Sung H., Ferlay J., Siegel R.L., Laversanne M., Soerjomataram I., Jemal A., Bray F. (2021). Global Cancer Statistics 2020: GLOBOCAN Estimates of Incidence and Mortality Worldwide for 36 Cancers in 185 Countries. CA Cancer J. Clin..

[2] Horváth A., Prekopp P., Polony G., Székely E., Tamás L., Dános K. (2021). Accuracy of the Preoperative Diagnostic Workup in Patients with Head and Neck Cancers Undergoing Neck Dissection in Terms of Nodal Metastases. Eur. Arch. Oto-Rhino-Laryngol..

[3] Hoang J.K., Vanka J., Ludwig B.J., Glastonbury C.M. (2013). Evaluation of Cervical Lymph Nodes in Head and Neck Cancer with CT and MRI: Tips, Traps, and a Systematic Approach. Am. J. Roentgenol..

[4] Audet N., Beasley N.J., MacMillan C., Jackson D.G., Gullane P.J., Kamel-Reid S. (2005). Lymphatic Vessel Density, Nodal Metastases, and Prognosis in Patients with Head and Neck Cancer. Arch. Otolaryngol. Head. Neck Surg..

[5] O’Brien C.J., Smith J.W., Soong S.-J., Urist M.M., Maddox W.A. (1986). Neck Dissection with and without Radiotherapy: Prognostic Factors, Patterns of Recurrence, and Survival. Am. J. Surg..

[6] de Bondt R.B.J., Nelemans P.J., Hofman P.A.M., Casselman J.W., Kremer B., van Engelshoven J.M.A., Beets-Tan R.G.H. (2007). Detection of Lymph Node Metastases in Head and Neck Cancer: A Meta-Analysis Comparing US, USgFNAC, CT and MR Imaging. Eur. J. Radiol..

[7] Schwartz L.H., Bogaerts J., Ford R., Shankar L., Therasse P., Gwyther S., Eisenhauer E.A. (2009). Evaluation of Lymph Nodes with RECIST 1.1. Eur. J. Cancer.

[8] Castelijns J.A., van den Brekel M.W. (2002). Imaging of Lymphadenopathy in the Neck. Eur. Radiol..

[9] Curtin H.D., Ishwaran H., Mancuso A.A., Dalley R.W., Caudry D.J., McNeil B.J. (1998). Comparison of CT and MR Imaging in Staging of Neck Metastases. Radiology.

[10] Parillo M., Quattrocchi C.C. (2025). Node Reporting and Data System 1.0 (Node-RADS) for the Assessment of Oncological Patients’ Lymph Nodes in Clinical Imaging. J. Clin. Med..

[11] Majumdar K.S., Rao V.U.S., Prasad R., Ramaswamy V., Sinha P., Subash A. (2020). Incidence of Micrometastasis and Isolated Tumour Cells in Clinicopathologically Node-Negative Head and Neck Squamous Cell Carcinoma. J. Maxillofac. Oral Surg..

[12] Yousem D.M., Som P.M., Hackney D.B., Schwaibold F., Hendrix R.A. (1992). Central Nodal Necrosis and Extracapsular Neoplastic Spread in Cervical Lymph Nodes: MR Imaging versus CT. Radiology.

[13] Kaji A.V., Mohuchy T., Swartz J.D. (1997). Imaging of Cervical Lymphadenopathy. Semin. Ultrasound CT MRI.

[14] Goldenberg D., Begum S., Westra W.H., Khan Z., Sciubba J., Pai S.I., Califano J.A., Tufano R.P., Koch W.M. (2008). Cystic Lymph Node Metastasis in Patients with Head and Neck Cancer: An HPV-Associated Phenomenon. Head Neck.

[15] Shah P.H., Karagianis A.G., Lester M.S., Paintal A.S., McComb E.N. (2022). Calcified Lymph Nodes in the Setting of Head and Neck Squamous Cell Carcinoma: A Predictor of HPV Positivity?. Clin. Imaging.

[16] Zumsteg Z.S., Luu M., Kim S., Tighiouart M., Mita A., Scher K.S., Lu D.J., Shiao S.L., Clair J.M.-S., Ho A.S. (2019). Quantitative Lymph Node Burden as a ‘Very-High-Risk’ Factor Identifying Head and Neck Cancer Patients Benefiting from Postoperative Chemoradiation. Ann. Oncol..

[17] Chen C.-C., Lin J.-C., Chen K.-W. (2015). Lymph Node Ratio as a Prognostic Factor in Head and Neck Cancer Patients. Radiat. Oncol..

[18] Sano D., Yabuki K., Takahashi H., Arai Y., Chiba Y., Tanabe T., Nishimura G., Oridate N. (2018). Lymph Node Ratio as a Prognostic Factor for Survival in Patients with Head and Neck Squamous Cell Carcinoma. Auris Nasus Larynx.

[19] de Ridder M., Marres C.C.M., Smeele L.E., van den Brekel M.W.M., Hauptmann M., Balm A.J.M., van Velthuysen M.L.F. (2016). A Critical Evaluation of Lymph Node Ratio in Head and Neck Cancer. Virchows Arch..

[20] Ji G.-W., Zhu F.-P., Zhang Y.-D., Liu X.-S., Wu F.-Y., Wang K., Xia Y.-X., Zhang Y.-D., Jiang W.-J., Li X.-C. (2019). A Radiomics Approach to Predict Lymph Node Metastasis and Clinical Outcome of Intrahepatic Cholangiocarcinoma. Eur. Radiol..

[21] Spuhler K.D., Ding J., Liu C., Sun J., Serrano-Sosa M., Moriarty M., Huang C. (2019). Task-Based Assessment of a Convolutional Neural Network for Segmenting Breast Lesions for Radiomic Analysis. Magn. Reson. Med..

[22] Chen L., Zhou Z., Sher D., Zhang Q., Shah J., Pham N.-L., Jiang S., Wang J. (2019). Combining Many-Objective Radiomics and 3D Convolutional Neural Network through Evidential Reasoning to Predict Lymph Node Metastasis in Head and Neck Cancer. Phys. Med. Biol..

[23] Seidler M., Forghani B., Reinhold C., Pérez-Lara A., Romero-Sanchez G., Muthukrishnan N., Wichmann J.L., Melki G., Yu E., Forghani R. (2019). Dual-Energy CT Texture Analysis with Machine Learning for the Evaluation and Characterization of Cervical Lymphadenopathy. Comput. Struct. Biotechnol. J..

[24] Sakr M. (2016). Cervical: Lymphadenopathy. Head and Neck and Endocrine Surgery.

[25] Brosch T., Saalbach A. (2018). Foveal Fully Convolutional Nets for Multi-Organ Segmentation. Proceedings of the Medical Imaging 2018: Image Processing, Houston, TX, USA, 2 March 2018.

[26] Rinneburger M., Carolus H., Iuga A.-I., Weisthoff M., Lennartz S., Hokamp N.G., Caldeira L., Shahzad R., Maintz D., Laqua F.C. (2023). Automated Localization and Segmentation of Cervical Lymph Nodes on Contrast-Enhanced CT Using a 3D Foveal Fully Convolutional Neural Network. Eur. Radiol. Exp..

[27] Ariji Y., Fukuda M., Nozawa M., Kuwada C., Goto M., Ishibashi K., Nakayama A., Sugita Y., Nagao T., Ariji E. (2021). Automatic Detection of Cervical Lymph Nodes in Patients with Oral Squamous Cell Carcinoma Using a Deep Learning Technique: A Preliminary Study. Oral Radiol..

[28] Payabvash S., Brackett A., Forghani R., Malhotra A. (2019). Differentiation of Lymphomatous, Metastatic, and Non-Malignant Lymphadenopathy in the Neck with Quantitative Diffusion-Weighted Imaging: Systematic Review and Meta-Analysis. Neuroradiology.

[29] Liao W., Luo X., Li L., Xu J., He Y., Huang H., Zhang S. (2025). Automatic Cervical Lymph Nodes Detection and Segmentation in Heterogeneous Computed Tomography Images Using Deep Transfer Learning. Sci. Rep..

[30] Pisani P., Airoldi M., Allais A., Aluffi Valletti P., Battista M., Benazzo M., Briatore R., Cacciola S., Cocuzza S., Colombo A. (2020). Metastatic Disease in Head & Neck Oncology. Acta Otorhinolaryngol. Ital..

[31] Al Hasan M.M., Ghazimoghadam S., Tunlayadechanont P., Mostafiz M.T., Gupta M., Roy A., Peters K., Hochhegger B., Mancuso A., Asadizanjani N. (2024). Automated Segmentation of Lymph Nodes on Neck CT Scans Using Deep Learning. J. Imaging Inform. Med..

[32] Zhou Z., Xue J., Wu Y., Mao J., Li C., Yu X., Ma C., Zhao G. (2025). Automated Detection of Metastatic Lymph Nodes in Head and Neck Malignant Tumors on High-Resolution MRI Images Using an Improved Convolutional Neural Network. Int. J. Med. Inform..

[33] Tan Y., Lu L., Bonde A., Wang D., Qi J., Schwartz L.H., Zhao B. (2018). Lymph Node Segmentation by Dynamic Programming and Active Contours. Med. Phys..

[34] Park J.-O., Nam I.-C., Kim C.-S., Park S.-J., Lee D.-H., Kim H.-B., Han K.-D., Joo Y.-H. (2022). Sex Differences in the Prevalence of Head and Neck Cancers: A 10-Year Follow-Up Study of 10 Million Healthy People. Cancers.

[35] Richard P., Sandison G., Johnson B., Liao J., Parvathaneni U. (2013). Incidence and Impact of Dental Amalgam Artifact on Radiation Therapy Target Volumes in Oropharyngeal and Oral Cavity Cancer. Int. J. Radiat. Oncol. Biol. Phys..

[36] Som P.M. (1992). Detection of Metastasis in Cervical Lymph Nodes: CT and MR Criteria and Differential Diagnosis. Am. J. Roentgenol..

[37] Nevens D., Vantomme O., Laenen A., Hermans R., Nuyts S. (2017). The Prognostic Value of Location and Size Change of Pathological Lymph Nodes Evaluated on CT-Scan Following Radiotherapy in Head and Neck Cancer. Cancer Imaging.

